# Genome Sequence of Hypervirulent *Aeromonas hydrophila* Strain HZAUAH

**DOI:** 10.1128/genomeA.00012-17

**Published:** 2017-03-16

**Authors:** Lin Teng, Limei Deng, Xingxing Dong, Shun Wei, Jinquan Li, Ningqiu Li, Yang Zhou

**Affiliations:** a Pearl River Fishery Research Institute, Chinese Academy of Fishery Sciences, Key Laboratory of Fishery Drug Development, Ministry of Agriculture, Key Laboratory of Aquatic Animal Immune Technology, Guangzhou, People's Republic of China; b Department of Animal Sciences, University of Florida, Gainesville, Florida, USA; c State Key Laboratory of Agricultural Microbiology, Huazhong Agricultural University, Wuhan, Hubei, People's Republic of China; d College of Fisheries, Huazhong Agricultural University, Wuhan, Hubei, People's Republic of China; e Department of Infectious Diseases and Pathology, University of Florida, Gainesville, Florida, USA

## Abstract

*Aeromonas hydrophila*, a zoonotic bacterium found in an expansive range of aquatic ecosystems, has been reported to cause severe diseases in fish, amphibians, reptiles, and mammals, including humans. Herein, we report the draft genome of the hypervirulent *A. hydrophila* strain HZAUAH isolated from a crucian in China.

## GENOME ANNOUNCEMENT

*Aeromonas hydrophila*, a Gram-negative and facultative anaerobic bacterium, is ubiquitous in a variety of aquatic environments (1) and is pathogenic to a range of animals, including fishes, reptiles, birds, and mammals (2). Diseases caused by this pathogen include fatal motile *Aeromonas* septicemia (MAS) in fish as well as gastroenteritis, septicemia, meningitis, and necrotizing fasciitis upon human infection (3–6). Historically, aquaculture outbreaks of MAS in fish have resulted in tremendous industrial economic loss in China (7) and the United States (8).

Prior studies by numerous investigative groups have revealed key *A. hydrophila* genetic traits which notably contribute to virulence, including genes encoding flagella, pili, secretion systems, and toxins (9–11). However, the molecular mechanisms by which* A. hydrophila* causes severe septicemia in fish remain to be established. Further characterization and evaluation of *A. hydrophila* virulence factors may support the development of countermeasures to combat MAS in fish aquaculture settings. *In vitro* analyses revealed the *A. hydrophila* strain HZAUAH, isolated from a crucian (*Carassius carassius*) with septicemia in 2015 from Hubei, China, to be more virulent than the well-established J-1 (10) strain (our unpublished data). In this study, we report the draft genome sequence of *A. hydrophila* strain HZAUAH.

*A. hydrophila* strain HZAUAH genomic DNA was extracted, followed by paired-end genomic DNA library construction using the Nano DNA HT sample preparation kit (Illumina) and sequencing by the HiSeq 2000 platform (Illumina, San Diego, CA, USA). Sequencing yielded a total number of 4,887,516 raw reads. Following quality trimming, *de novo* assembly was conducted using SOAP*denovo* version 2.04 (12), which generated 32 contigs with a total size of 5,035,588 bp (*N*_50_, 471,075 bp) and a G+C content of 60.86%. Depth of coverage was approximately 108-fold.

After assembly, initial analysis was performed using PATRIC (13) (https://www.patricbrc.org/), which annotated 4,806 coding sequences (CDSs), 3 rRNA genes, and 95 tRNA genes and determined the *A. hydrophila* multilocus sequence type (ST) to be ST251. The genome encodes previously described virulence factors, such as cytotoxic enterotoxin (AerA), heat-stable cytotonic enterotoxin (Ast), extracellular hemolysin (AHH1), hemolysin (HlyA), hemolysin III, thermostable hemolysin (TH), RtxA, AroA, DNA adenine methyltransferase (Dam), elastase (AhpB), enolase (Eno), extracellular protease (EprA1), glucose-inhibited division protein A (GidA), phospholipase A1 (PLA), phospholipase C (PLC), exoribonuclease R (VacB), serine protease (SerA), ToxR-regulated lipoprotein (TagA), UDP *N*-acetylgalactosamine 4-epimerase (Gne), UDP-galactose-4-epimerase (GalE), UDP-glucose pyrophosphorylase (GalU), and nuclease (Ahn) (9, 10). The PHAge search tool (PHAST) (14) predicted four prophage regions, including two intact prophage regions (one with 30.2 kb, 36 CDSs, and a G+C content of 56.56%, and one with 43.6 kb, 51 CDSs, and a >G+C content of 58.28%) and one incomplete prophage region (17.5 kb, 20 CDSs, and >G+C content of 50.67%).

### Accession number(s).

This whole-genome shotgun project has been deposited in GenBank under the accession no. MRDF00000000. The version described in this paper is the first version.
